# Perioperative Hypotension in Patients Undergoing Orthopedic Upper Extremity Surgery with Dexmedetomidine Sedation: A Retrospective Study

**DOI:** 10.3390/jpm13121658

**Published:** 2023-11-28

**Authors:** Sujin Baek, Jiyong Lee, Yong Sup Shin, Yumin Jo, Juyeon Park, Myungjong Shin, Chahyun Oh, Boohwi Hong

**Affiliations:** 1 Department of Anesthesiology and Pain Medicine, Chungnam National University Hospital, Daejeon 35015, Republic of Koreagdragonlee@cnuh.co.kr (J.L.); ysshin@cnu.ac.kr (Y.S.S.); parkjuyeon@cnuh.co.kr (J.P.); audwhd309@cnuh.co.kr (M.S.); 2 Department of Anesthesiology and Pain Medicine, College of Medicine, Chungnam National University, Daejeon 34134, Republic of Korea

**Keywords:** dexmedetomidine, sedation, hypotension, regional anesthesia, peripheral nerve block, orthopedic surgery

## Abstract

(1) Background: limited data exist regarding the occurrence of hypotension associated with dexmedetomidine use and its risk factors in the context of intraoperative sedation for patients receiving peripheral nerve blocks. (2) Method: This single-center retrospective study assessed the incidence of hypotension in patients undergoing orthopedic upper extremity surgery with brachial plexus blockade. Patients were classified into three groups: group N (non-sedated), group M (midazolam), and group D (dexmedetomidine), based on their primary intraoperative sedative use. The primary outcome was the incidence of perioperative hypotension, defined as systolic blood pressure (SBP) < 90 mmHg or mean blood pressure (MBP) < 60 mmHg, at a minimum of two recorded time points during the intraoperative period and post-anesthesia care unit stay. Multivariable logistic models for the occurrence of hypotension were constructed for the entire cohort and group D. (3) Results: A total of 2152 cases (group N = 445, group M = 678, group D = 1029) were included in the analysis. The odds ratio for the occurrence of hypotension in group D was 5.68 (95% CI, 2.86 to 11.28) compared with group N. Concurrent use of a beta blocker, longer duration of surgery, and lower preoperative SBP and higher preoperative heart rate were identified as significant risk factors. (4) Conclusions: the increased risk of hypotension and the associated factors should be taken into account before using dexmedetomidine in these cases.

## 1. Introduction

Dexmedetomidine, a highly selective alpha-2 adrenergic agonist, possesses both hypnotic and analgesic properties, making it a widely used agent for perioperative sedation. Notably, it can provide an appropriate level of sedation without inducing respiratory depression [1,2]. Moreover, it has found extensive application in both intensive care units (ICU) and perioperative settings for its capacity to maintain optimal conscious sedation and its potential in preventing delirium [3,4]. However, it is important to note that dexmedetomidine can lead to hemodynamic compromise such as hypotension and bradycardia, which are well documented in ICU settings [4]. Preexisting low blood pressure, history of coronary artery disease, and advanced age were identified as independent risk factors for dexmedetomidine-associated hypotension in critically ill patients [5,6].

Dexmedetomidine is also commonly used for patients undergoing orthopedic surgery with regional anesthesia. Thanks to its ability to prolong the analgesic effect of regional blockade and reduce postoperative opioid consumption, dexmedetomidine is considered a valuable sedative agent following peripheral nerve blockade [7,8]. Although these benefits of dexmedetomidine have been widely recognized, its potential negative impact on hemodynamics, particularly in the context of intraoperative sedation for patients receiving peripheral nerve blocks, has gained less research attention. This study, therefore, aimed to assess the incidence and risk factors of dexmedetomidine-associated hypotension in patients undergoing orthopedic upper extremity surgery with brachial plexus blockade (BPB).

## 2. Materials and Methods

This retrospective observational study was approved by the Institutional Review Board of Chungnam National University Hospital (CNUH 2020-07-053) on 14 August 2020, and the trial was registered at the Clinical Research Information Service, a clinical trial registry in South Korea (CRIS, identifier: KCT0005825). Informed consent was waived due to the retrospective design of the study. This manuscript adheres to the applicable STROBE (Strengthening the Reporting of Observational Studies in Epidemiology) guidelines [9].

We conducted a retrospective review of electronic medical records for all patients who underwent orthopedic upper extremity surgery under BPB between January 2015 and December 2019. In order to assess the relative risk of hypotension, our analysis encompassed not only patients sedated with dexmedetomidine but also non-sedated patients and those sedated with midazolam. In this analysis, we extracted intraoperative and post-anesthesia care unit (PACU) vital signs, comprising up to 40 data points at 5 min intervals, resulting in a total recording duration of up to 200 min per patient. Patients who underwent multiple surgeries during the study period were considered eligible and treated as independent cases. Exclusion criteria for our study encompassed cases in a sitting position during surgery and those with insufficient data. Insufficient data was defined as instances where the retrieved vital sign recording was less than 75% of the entire duration of the patient’s stay in the operating room and PACU.

### 2.1. Anesthetic Protocol

Patients were continuously monitored from the moment they entered the operating room to their discharge from PACU. Monitoring included noninvasive intermittent blood pressure measurements using a cuff, a 3-lead electrocardiogram, and pulse oximetry. Blood pressure was assessed at 5 min intervals, primarily in the non-surgical arm.

All BPBs were performed under ultrasound guidance using either 0.375% to 0.5% ropivacaine or a 1:1 mixture of 0.75% ropivacaine and 1% lidocaine, without any addition of adjuvants.

Sedation was commenced after verifying adequate onset of the regional blockade in the surgical area. The choice of sedatives was at the discretion of the attending anesthesiologist. Patients were classified into three groups: group N (non-sedated), group M (midazolam), and group D (dexmedetomidine), based on their primary intraoperative sedative use. Patients in group M received intermittent bolus doses of midazolam, with a maximum of 5 mg at a time, as needed. In group D, patients received continuous administration of dexmedetomidine via an infusion pump. A typical regimen involved an initial loading dose of 1 μg/kg (based on ideal body weight) administered over 10 min, followed by a continuous maintenance infusion at a rate of 0.3–1 μg/kg/hr. The administration of the loading dose varied depending on the attending physician’s discretion. Given the gradual onset of sedative effect, small doses of supplementary midazolam were employed in group D when rapid adjustments of the sedation level were required. All dosages were titrated to achieve moderate-to-deep sedation (Richmond Agitation and Sedation Scale: −3 to −4). Supplementary opioid (fentanyl, 20–50 μg) or small doses of propofol (10–30 mg) were allowed to relieve intolerable discomfort or to control persistent or sudden movement of the patient during the procedure.

Perioperative hypotensive events were managed by administering bolus doses of vasoactive drugs, such as phenylephrine 50 μg or ephedrine 5 mg, or through fluid challenges as deemed necessary.

After the completion of surgery, patients were transferred to the PACU and monitored until the modified Aldrete score reached at least 9. If a patient did not meet the discharge criteria, the attending anesthesiologist reassessed the patient and made the decision regarding their discharge from PACU.

### 2.2. Outcome Measurements and Covariates

The primary outcome of the study was the incidence of perioperative hypotension, which encompassed both intraoperative and postoperative occurrences in the PACU. Hypotension was defined as systolic blood pressure (SBP) < 90 mmHg or mean blood pressure (MBP) < 60 mmHg at a minimum of two recorded time points, whether consecutive or not. Secondary outcomes included the incidences of SBP < 90 mmHg, MBP < 60 mmHg, bradycardia (heart rate (HR) < 50 beats per minute), the onset time of a hypotensive event (the first time point when the criteria for a hypotensive event were met), and the duration of PACU stay.

Covariates extracted from the electronic medical records included the following: demographic information (age, sex, weight, height, body mass index); comorbid conditions (hypertension, diabetes mellitus, coronary artery disease); regular antihypertensive medications (calcium channel blocker, angiotensin-converting enzyme inhibitors/angiotensin receptor blocker, beta blocker, diuretics); duration of surgery (covering the entire duration in the operating room); and the latest preoperative vital signs.

### 2.3. Statistical Analysis

The sample size was determined based on the available data from patients who underwent upper extremity orthopedic surgery under BPB at our institution during the study period, rather than through a priori power analysis. All statistical analyses were conducted using R software (version 4.2.2, R Project for Statistical Computing, Vienna, Austria). The distribution of continuous variables was assessed using the Shapiro–Wilk test, with non-normally distributed variables presented as median (1Q, 3Q), and group comparisons were performed using the Kruskal–Wallis test. Categorical data were compared using the χ^2^-squared test or Fisher’s exact test, as appropriate, with the results expressed as number (%).

The risk of hypotension was assessed in both the entire cohort and group D. Covariates that exhibited a *p* value < 0.1 in univariable analysis, along with clinically relevant variables (hypertension, coronary artery disease), were incorporated into the subsequent multivariable logistic model. To enhance interpretability, preoperative vital signs were rounded to the nearest 10 and divided by 10 before conducting logistic regression. In the multivariable models, preoperative SBP was chosen over diastolic blood pressure (DBP) due to their correlation (multicollinearity). In addition, the use of supplementary midazolam was included in the subgroup (group D) multivariable model. Multicollinearity was assessed using the variance inflation factor, where a value greater than 5 indicates the presence of multicollinearity. The models’ fit and discriminatory power were assessed using the Hosmer–Lemeshow test and by calculating the area under the receiver operating characteristic curve (AUROC), respectively. A two-tailed *p* value < 0.05 was considered statistically significant for all calculations.

## 3. Results

**Figure 1:**
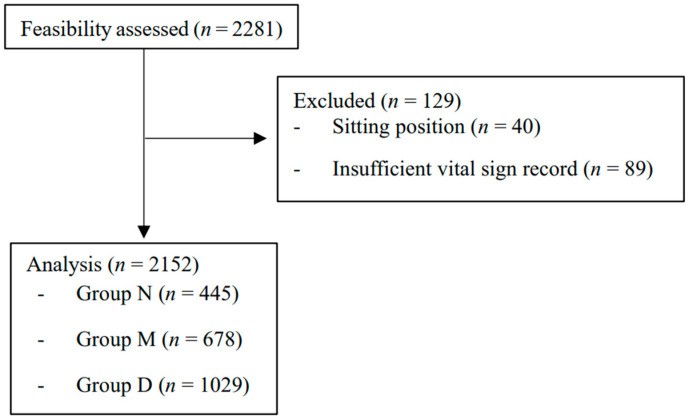
Patient flow diagram. Groups N, M, and D represent non-sedated, midazolam, and dexmedetomidine subjects, respectively.

A total of 2281 patients underwent upper extremity orthopedic surgery under BPB during the research period. Of these, 40 cases in the sitting position and 89 cases with insufficient record length were excluded. The remaining 2152 cases (group N = 445, group M = 678, group D = 1029) were included in the analysis (Figure 1). There were 354, 343, and 343 missing values in the latest preoperative SBP, DBP, and HR data. The demographic and clinical characteristics of the included cases are summarized in Table 1.

**Figure 2:**
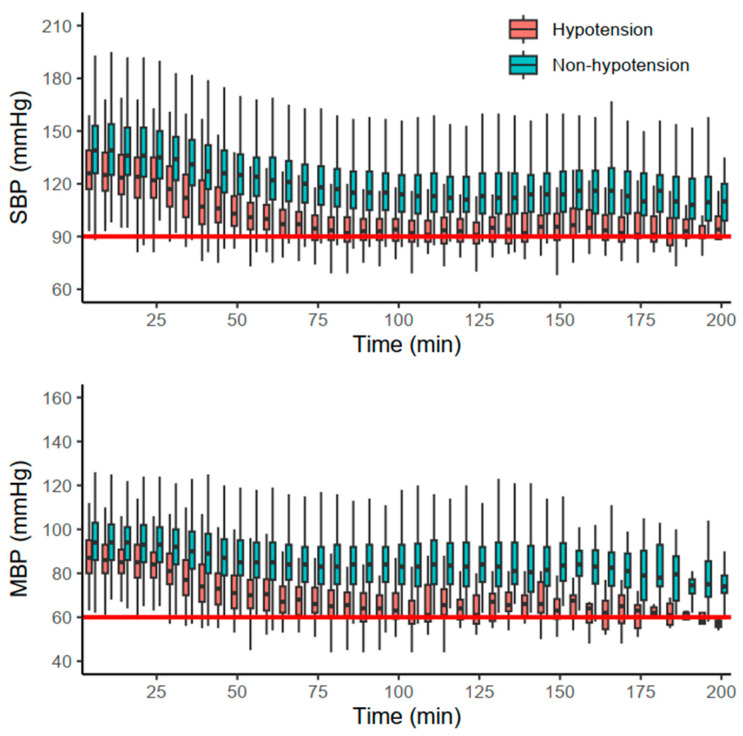
Hemodynamic changes in group D stratified by the occurrence of hypotension. Red and blue boxplots indicate cases with and without hypotension, respectively. The red solid horizontal lines indicate the thresholds of systolic blood pressure (SBP) and mean blood pressure (MBP).

The primary and secondary outcomes are summarized in Table 2. Hypotensive events were more frequent in group D (13.8%) than in group N (2.9%) and group M (0.9%) (*p* < 0.001). The majority of hypotensive events occurred after the first hour (78.2%) in group D, whereas the onsets in other groups were evenly distributed (*p* = 0.037). Additionally, each of the other hemodynamic events (SBP < 90 mmHg, MBP < 60 mmHg, bradycardia) also occurred more frequently in group D (*p* < 0.001). Hemodynamic changes in group D are depicted in Figure 2. Furthermore, the duration of PACU stay was significantly longer in group D (36.0 [27.0, 49.0] min) than in the other groups (*p* < 0.001).

The univariable and multivariable analysis of hypotension in the entire cohort is presented in Table 3. The odds ratio (OR) for the occurrence of hypotension in group D was 5.68 (95% CI, 2.86 to 11.28) compared with group N. Advanced age (*p* = 0.033), concurrent use of a beta blocker (*p* = 0.019), longer duration of surgery (*p* = 0.001), and lower preoperative SBP (*p* <0.001) and higher preoperative HR (*p* = 0.001) were identified as significant risk factors. The model showed no evidence of poor fit (*p* = 0.700), with an AUROC of 0.828 (95% CI, 0.797 to 0.859).

The univariable and multivariable analysis of hypotension in group D is presented in Table 4. Concurrent use of a beta blocker (*p* = 0.023), longer duration of surgery (*p* = 0.020), and lower preoperative SBP (*p* <0.001) and higher preoperative HR (*p* = 0.005) were identified as significant risk factors. The model showed no evidence of poor fit (*p* = 0.967), with an AUROC of 0.721 (95% CI, 0.673 to 0.769).

## 4. Discussion

The current study revealed that the incidence of hypotension with the use of dexmedetomidine for sedation during orthopedic upper extremity surgery under BPB was 13.8%. The risk of hypotension was significantly higher with the use of dexmedetomidine, with an OR of 5.68, in comparison to patients who were not sedated. Notably, approximately 80% of hypotensive events occurred after the first hour of the surgery in patients sedated with dexmedetomidine. Additionally, concurrent use of a beta blocker, longer duration of surgery, and lower preoperative SBP and higher preoperative HR were significant risk factors for hypotension in both the entire cohort and group D. As far as we know, this is the largest study assessing dexmedetomidine-associated hypotension in orthopedic upper extremity surgery.

There is abundant evidence suggesting that even brief episodes of intraoperative hypotension can be detrimental during non-cardiac surgery [10]. For instance, a large retrospective cohort study involving 33,330 non-cardiac surgeries reported that any duration of mean arterial pressure below 55 mmHg was linked to adverse outcomes [11], underscoring the significance of vigilant intraoperative blood pressure management. Therefore, careful consideration is essential when choosing perioperative sedative agents, particularly for vulnerable patients who are susceptible to organ damage.

Dexmedetomidine is a potent α2-adrenoreceptor agonist that exerts sympatholytic effects by inhibiting the release of norepinephrine in sympathetic nerve endings. The cardiovascular effects of dexmedetomidine are characterized by transient hypertension followed by subsequent hypotension [12]. Initially, hypertension is attributed to vasoconstriction of vascular smooth muscle induced by the stimulation of peripheral α-2B receptors. Subsequent hypotension occurs when the vasodilatory effects of the central α-2A receptors become predominant. This biphasic hemodynamic change appears to partially account for the predominant occurrence of hypotension in the later stages of surgery in group D.

Previous studies that assessed the occurrence of dexmedetomidine-associated hypotension reported various incidences ranging from about 20 to 70% [4,5,6,13,14,15], often exceeding the 13.8% of incidence found in the current study. As the previous studies were mostly conducted in ICU settings, the higher incidences of hypotension seem to result from the underlying illness of the patients and prolonged infusion duration, which was also a significant risk factor (i.e., longer duration of surgery) in our study.

A recent study conducted in a setting similar to ours, which involved orthopedic upper extremity surgery under BPB, employed both absolute (SBP of 90 mmHg) and relative (30% decrease from baseline) thresholds to define hypotension [16]. The study reported an incidence of 14.7%, identifying female sex and obesity as significant risk factors. While the incidence of hypotension was similar to our finding, the discrepancy in the definition of hypotension poses challenges in directly comparing these studies. While adopting an individualized approach with a relative threshold for blood pressure is a reasonable practice, there is conflicting evidence on this matter. Among the various pieces of evidence, a large-scale retrospective study specifically focused on comparing intraoperative MBP below an absolute threshold of 65 mmHg and a relative threshold of 20% in terms of their impact on the occurrence of myocardial and kidney injury [17]. This study concluded that relative thresholds were no more effective than those based on absolute criteria. Furthermore, given that most patients experience heightened stress before surgery, establishing a true baseline blood pressure, especially in a retrospective approach, can be challenging. Given this context, we chose to use absolute thresholds for defining hypotension in our study.

In our current study, we have identified significant factors contributing to dexmedetomidine-associated hypotension. Firstly, the concurrent use of a beta blocker seems to be a plausible risk factor due to its negative chronotropic effect. However, the evidence supporting this finding is currently limited and weak [18] and requires further validation. Notably, a randomized trial assessing the safety and tolerability of intranasal dexmedetomidine found no significant effect of beta blockers on the occurrence of hypotension [19]. Secondly, the relationship between preoperative blood pressure and dexmedetomidine-induced hypotension aligns with previous research [5,6,20]. While a hypertensive state is generally not desired, it may offer a greater safety margin when dealing with dexmedetomidine-associated hypotension. Thirdly, what is intriguing is that our findings indicate that a higher preoperative heart rate, rather than a lower one, is a significant risk factor. One possible explanation could be a high baseline heart rate combined with a lower baseline stroke volume. However, currently, there is no clear explanation for this phenomenon.

Dexmedetomidine is a commonly chosen sedative agent for orthopedic procedures not only due to its favorable respiratory profile but also for its ability to extend the duration of spinal or peripheral nerve blocks and reduce postoperative opioid consumption [7,8,21,22,23]. This favorable effect may be dose-dependent, especially in the case of peripheral blockades. In our previous study, we observed that dexmedetomidine sedation (at a mean dose of 1.6 μg/kg) significantly extended the duration of the blockade’s analgesic effects by approximately 3 h [8]. However, we could not replicate this result in our subsequent study, where we used slightly reduced doses (mean of 1.1–1.2 μg/kg) [24]. In the meantime, in a recent cohort study, it was found that the risk of postoperative hypotension increased to about twice the baseline when the total intraoperative dexmedetomidine dose exceeded 50 μg [20]. This suggests that even a 1 μg/kg loading dose alone can lead to intraoperative hypotension. These contrasting effects of dexmedetomidine on postoperative analgesia and hypotension should be taken into consideration when planning intraoperative sedation.

Furthermore, it is crucial to emphasize the significantly increased risk of bradycardia (20.3% incidence) associated with the administration of dexmedetomidine. Although our study did not observe fatal hemodynamic compromises resulting from these events, it is important to acknowledge that dexmedetomidine-induced bradycardia could, on occasion, progress to more extreme outcomes such as asystole. In a previous randomized trial, hypoxemia and bradycardia events were more frequently observed in patients receiving opioid-free anesthesia with dexmedetomidine compared with those receiving remifentanil [25]. This trial was terminated prematurely due to severe cases of bradycardia. Intriguingly, these severe instances were predominantly linked to exacerbated vagal stimulus during carbon dioxide insufflation. Therefore, a meticulous evaluation of the risk of bradycardia is imperative before using dexmedetomidine, particularly in situations where there is a potential for exacerbated vagal response.

The extended PACU stay observed in group D is consistent with findings from previous studies, even though the clinical contexts in which dexmedetomidine was administered differed. A large-scale retrospective study, involving 130,854 cases of diverse ambulatory procedures with various anesthesia types (including general, regional, neuraxial, and monitored anesthesia care), revealed a prolonged PACU stay in patients who received intraoperative dexmedetomidine [26]. This extension was found to be dose-dependent and influenced by the type of anesthesia used. The underlying reason for this prolongation was attributed to the extended half-life of dexmedetomidine and its sympatholytic effects, which can result in excessive sedation and cardiovascular complications in the PACU. Although a definitive causal analysis was not possible, this could also account for the results of the current study. Conversely, a recent meta-analysis, which included randomized trials conducted with patients undergoing general anesthesia, presented conflicting results [27]. In this study, the use of dexmedetomidine did not lead to a prolonged PACU stay, while reducing various postoperative events and issues related to emergence. Consequently, it appears that the impact of dexmedetomidine on PACU stay is intricately linked to a range of clinical factors, including the type of anesthesia, dosage of the drug, and concurrent medications such as opioids.

The present study has several limitations. Firstly, due to its retrospective nature, detailed information regarding the dosing of dexmedetomidine, rather than the general protocol, was not available. Although the dose-dependent effect of dexmedetomidine on hypotension has not been consistent [5,14], confounding effects due to this issue cannot be excluded. Additionally, the depth of sedation was not strictly controlled or reported, and this also introduces a confounding effect. Secondly, detailed information regarding the use of vasopressors and supplemental opioids was not included in the analysis. While we anticipate that the use of either agent would be infrequent in orthopedic upper extremity surgery under BPB, we cannot rule out potential confounding effects, particularly considering the synergistic hemodynamic effect of opioids when used in conjunction with other sedatives. Lastly, we did not assess the occurrence of clinically relevant outcomes, such as cardiovascular and renal complications. Therefore, the clinical relevance of dexmedetomidine-associated hypotension cannot be definitively concluded within the scope of the current study.

## 5. Conclusions

The incidence of hypotension associated with the use of dexmedetomidine for sedation during orthopedic upper extremity surgery under BPB was 13.8%, which was significantly higher than in patients who received midazolam or were not sedated. The increased risk of hypotension and the associated factors should be taken into account before using dexmedetomidine in these cases.

## Figures and Tables

**Table 1:** Demographic and clinical characteristics.

	Group N (*n* = 445)	Group M (*n* = 678)	Group D (*n* = 1029)	*p*
Age (yr)	58.0 (46.0, 70.0)	55.0 (38.0, 66.0)	54.0 (37.0, 66.0)	<0.001
Sex (male)	258 (57.5)	351 (51.8)	540 (52.5)	0.146
Weight (kg)	62.3 (55.0, 71.6)	62.5 (55.0, 70.8)	64.0 (56.4, 73.2)	0.026
Height (cm)	163.3 (154.7, 170.7)	162.1 (155.0, 170.0)	163.0 (155.3, 171.4)	0.392
BMI (kg/m^2^)	23.8 (21.6, 26.0)	23.9 (21.9, 26.0)	24.2 (22.1, 26.5)	0.057
ASA				<0.001
1	93 (20.7)	142 (20.9)	230 (22.4)	
2	250 (56.2)	462 (68.1)	659 (64.0)	
≥3	102 (22.9)	74 (10.9)	140 (13.6)	
Comorbidities				
HTN	34 (7.6)	56 (8.3)	91 (8.8)	0.736
DM	50 (11.2)	53 (7.8)	85 (8.3)	0.105
CAD	13 (2.9)	8 (1.2)	15 (1.5)	0.064
Antihypertensive medications				
CCB	38 (8.5)	56 (8.3)	81 (7.9)	0.902
ARB/ACEi	33 (7.4)	57 (8.4)	85 (8.3)	0.820
BB	5 (1.1)	8 (1.2)	27 (2.6)	0.042
Diuretics	23 (5.2)	31 (4.6)	30 (2.9)	0.068
Duration of surgery (hr)	1.2 (1.0, 1.6)	1.2 (1.0, 1.4)	1.3 (1.1, 1.7)	<0.001
Supplementary midazolam	0 (0.0)	0 (0.0)	205 (19.9%)	<0.001
Preoperative vital signs				
SBP (mmHg)	126.0 (110.0, 139.5)	120.0 (110.0, 132.0)	124.0 (113.0, 137.0)	0.001
DBP (mmHg)	77.0 (68.0, 82.0)	72.0 (65.0, 80.0)	73.0 (66.0, 81.0)	<0.001
HR (beats per minute)	70.0 (62.0, 77.0)	66.0 (60.0, 75.0)	67.0 (61.0, 75.0)	<0.001

Values are number (%) or median (1Q, 3Q). Groups N, M, and D represent non-sedated, midazolam, and dexmedetomidine subjects, respectively. Abbreviations: BMI, body mass index; ASA, American Society of Anesthesiologists physical status; HTN, hypertension; DM, diabetes mellitus; CAD, coronary artery disease; CCB, calcium channel blocker; ARB/ACEi, angiotensin-converting enzyme inhibitors/angiotensin receptor blocker; BB, beta blocker; SBP, systolic blood pressure; DBP, diastolic blood pressure; HR, heart rate.

**Table 2:** Crude incidences of the hemodynamic events and secondary outcomes.

	Group N (*n* = 445)	Group M (*n* = 678)	Group D (*n* = 1029)	*p*
Hypotension	13 (2.9)	6 (0.9)	142 (13.8)	<0.001
Onset time (min)	60.0 (50.0, 95.0)	67.5 (35.0, 85.0)	80.0 (60.0, 95.0)	0.275
Onset ≥ 1 h	6 (50.0)	3 (50.0)	111 (78.2)	0.037
SBP < 90 mmHg	11 (2.5)	5 (0.7)	130 (12.6)	<0.001
MBP < 60 mmHg	6 (1.3)	3 (0.4)	54 (5.2)	<0.001
HR < 50 beats per minute	16 (3.6)	61 (9.0)	209 (20.3)	<0.001
PACU stay (min)	24.0 (20.0, 31.0)	27.0 (22.0, 35.0)	36.0 (27.0, 49.0)	<0.001

Values are number (%) or median (1Q, 3Q). Groups N, M, and D represent non-sedated, midazolam, and dexmedetomidine subjects, respectively. Abbreviations: SBP, systolic blood pressure; MBP, mean blood pressure; HR, heart rate; PACU, post-anesthesia care unit.

**Table 3:** Univariable and multivariable logistic regression for the incidence of hypotension in the entire cohort.

	Univariable Analysis			Multivariable Analysis		
	OR	95% CI	*p*	OR	95% CI	*p*
Group						
N *						
M	0.30	0.11, 0.79	0.015	0.30	0.10, 0.89	0.030
D	5.32	2.98, 9.50	<0.001	5.68	2.86, 11.28	<0.001
Age (yr)	1.01	1.00, 1.02	0.065	1.01	1.00, 1.03	0.033
Sex (male)	0.67	0.48, 0.92	0.014	0.94	0.55, 1.62	0.825
Weight (kg)	0.99	0.97, 1.00	0.037	1.01	0.99, 1.03	0.503
Height (cm)	0.98	0.96, 0.99	0.003	0.98	0.95, 1.02	0.349
BMI (kg/m^2^)	0.99	0.94, 1.03	0.645			
ASA (≥3)	1.49	0.98, 2.21	0.063	1.21	0.67, 2.19	0.527
HTN	1.22	0.69, 2.04	0.478	1.01	0.51, 1.98	0.978
DM	1.00	0.54, 1.70	0.985			
CAD	2.03	0.68, 4.85	0.183	2.48	0.55, 11.11	0.235
CCB	1.47	0.85, 2.40	0.160			
ARB/ACEi	1.18	0.65, 1.99	0.575			
BB	3.20	1.35, 6.74	0.010	3.29	1.22, 8.90	0.019
Diuretics	1.13	0.47, 2.33	0.766			
Duration of surgery (hr)	2.12	1.61, 2.80	<0.001	1.83	1.30, 2.59	0.001
Preoperative vital signs						
SBP (10 mmHg) **	0.74	0.66, 0.83	<0.001	0.62	0.55, 0.72	<0.001
DBP (10 mmHg) **	0.70	0.59, 0.81	<0.001			
HR (10 beats per minute) **	1.26	1.09, 1.46	0.002	1.33	1.13, 1.56	0.001

* Reference category. ** To enhance interpretability, values were rounded to the nearest 10 and divided by 10. Groups N, M, and D represent non-sedated, midazolam, and dexmedetomidine subjects, respectively. Abbreviations: OR, odds ratio; CI, confidence interval; BMI, body mass index; ASA, American Society of Anesthesiologists physical status; HTN, hypertension; DM, diabetes mellitus; CAD, coronary artery disease; CCB, calcium channel blocker; ARB/ACEi, angiotensin-converting enzyme inhibitors/angiotensin receptor blocker; BB, beta blocker; SBP, systolic blood pressure; DBP, diastolic blood pressure; HR, heart rate.

**Table 4:** Univariable and multivariable logistic regression for the incidence of hypotension in group D.

	Univariable Analysis			Multivariable Analysis		
	OR	95% CI	*p*	OR	95% CI	*p*
Age (yr)	1.01	1.00, 1.02	0.024	1.01	1.00, 1.03	0.095
Sex (male)	0.64	0.45, 0.92	0.014	0.88	0.49, 1.57	0.666
Weight (kg)	0.98	0.97, 1.00	0.032	1.01	0.99, 1.04	0.216
Height (cm)	0.97	0.96, 0.99	0.002	0.98	0.94, 1.01	0.219
BMI (kg/m^2^)	0.99	0.94, 1.04	0.685			
ASA (≥3)	1.61	1.00, 2.52	0.052	1.24	0.65, 2.38	0.520
HTN	1.15	0.61, 2.03	0.651	0.92	0.44, 1.92	0.822
DM	0.82	0.39, 1.56	0.562			
CAD	1.57	0.36, 5.03	0.506	0.84	0.09, 8.18	0.879
CCB	1.75	0.96, 3.02	0.065	1.63	0.81, 3.29	0.175
ARB/ACEi	1.26	0.66, 2.23	0.466			
Beta blocker	2.74	1.11, 6.19	0.030	3.31	1.18, 9.30	0.023
Diuretics	1.59	0.58, 3.71	0.343			
Duration of surgery (hr)	1.58	1.14, 2.18	0.007	1.56	1.07, 2.27	0.020
Supplementary midazolam	0.89	0.55, 1.38	0.601	0.94	0.56, 1.58	0.829
Preoperative vital signs						
SBP (10 mmHg) *	0.71	0.62, 0.81	<0.001	0.63	0.54, 0.73	<0.001
DBP (10 mmHg) *	0.68	0.57, 0.80	<0.001			
HR (10 beats per minute) *	1.24	1.05, 1.45	0.011	1.29	1.08, 1.54	0.005

* To enhance interpretability, values were rounded to the nearest 10 and divided by 10. Abbreviations: OR, odds ratio; CI, confidence interval; BMI, body mass index; ASA, American Society of Anesthesiologists physical status; HTN, hypertension; DM, diabetes mellitus; CAD, coronary artery disease; CCB, calcium channel blocker; ARB/ACEi, angiotensin-converting enzyme inhibitors/angiotensin receptor blocker; BB, beta blocker; SBP, systolic blood pressure; DBP, diastolic blood pressure; HR, heart rate.

## Data Availability

The data presented in this study are available on reasonable request from the corresponding author.

## References

[1] Lee S. (2019). Dexmedetomidine: Present and future directions. Korean J. Anesthesiol..

[2] Kim K.H. (2014). Safe Sedation and Hypnosis using Dexmedetomidine for Minimally Invasive Spine Surgery in a Prone Position. Korean J. Pain.

[3] Ng K.T., Shubash C.J., Chong J.S. (2019). The effect of dexmedetomidine on delirium and agitation in patients in intensive care: Systematic review and meta-analysis with trial sequential analysis. Anaesthesia.

[4] Jakob S.M., Ruokonen E., Grounds R.M., Sarapohja T., Garratt C., Pocock S.J., Bratty J.R., Takala J., Dexmedetomidine for Long-Term Sedation Investigators (2012). Dexmedetomidine vs. midazolam or propofol for sedation during prolonged mechanical ventilation: Two randomized controlled trials. JAMA.

[5] Ice C.J., Personett H.A., Frazee E.N., Dierkhising R.A., Kashyap R., Oeckler R.A. (2016). Risk Factors for Dexmedetomidine-Associated Hemodynamic Instability in Noncardiac Intensive Care Unit Patients. Anesth. Analg..

[6] Gerlach A.T., Blais D.M., Jones G.M., Burcham P.K., Stawicki S.P., Cook C.H., Murphy C.V. (2016). Predictors of dexmedetomidine-associated hypotension in critically ill patients. Int. J. Crit. Illn. Inj. Sci..

[7] Kang R., Choi J.W., Sung K.S., Wi W., Hahm T.S., Cho H.S., Yang M.K., Ko J.S. (2020). Effect of Intraoperative Sedation with Dexmedetomidine Versus Propofol on Acute Postoperative Pain Following Major Foot Surgery under Popliteal Sciatic Nerve Block: A Randomized Controlled Trial. J. Clin. Med..

[8] Hong B., Jung C., Jo Y., Kang H., Chung W., Kim Y.H., Lim C., Ko Y. (2019). Sedation with dexmedetomidine prolongs the analgesic duration of brachial plexus block: A randomised controlled trial. Anaesth. Crit. Care Pain Med..

[9] von Elm E., Altman D.G., Egger M., Pocock S.J., Gøtzsche P.C., Vandenbroucke J.P. (2007). The Strengthening the Reporting of Observational Studies in Epidemiology (STROBE) statement: Guidelines for reporting observational studies. Lancet.

[10] Sessler D.I., Bloomstone J.A., Aronson S., Berry C., Gan T.J., Kellum J.A., Plumb J., Mythen M.G., Grocott M.P.W., Edwards M.R. (2019). Perioperative Quality Initiative consensus statement on intraoperative blood pressure, risk and outcomes for elective surgery. Br. J. Anaesth..

[11] Walsh M., Devereaux P.J., Garg A.X., Kurz A., Turan A., Rodseth R.N., Cywinski J., Thabane L., Sessler D.I. (2013). Relationship between intraoperative mean arterial pressure and clinical outcomes after noncardiac surgery: Toward an empirical definition of hypotension. Anesthesiology.

[12] Talke P., Anderson B.J. (2018). Pharmacokinetics and pharmacodynamics of dexmedetomidine-induced vasoconstriction in healthy volunteers. Br. J. Clin. Pharmacol..

[13] Gerlach A.T., Dasta J.F., Steinberg S., Martin L.C., Cook C.H. (2009). A new dosing protocol reduces dexmedetomidine-associated hypotension in critically ill surgical patients. J. Crit. Care.

[14] Jones G.M., Murphy C.V., Gerlach A.T., Goodman E.M., Pell L.J. (2011). High-dose dexmedetomidine for sedation in the intensive care unit: An evaluation of clinical efficacy and safety. Ann. Pharmacother..

[15] Erdman M.J., Doepker B.A., Gerlach A.T., Phillips G.S., Elijovich L., Jones G.M. (2014). A comparison of severe hemodynamic disturbances between dexmedetomidine and propofol for sedation in neurocritical care patients. Crit. Care Med..

[16] Doo A.R., Lee H., Baek S.J., Lee J. (2021). Dexmedetomidine-induced hemodynamic instability in patients undergoing orthopedic upper limb surgery under brachial plexus block: A retrospective study. BMC Anesthesiol..

[17] Salmasi V., Maheshwari K., Yang D., Mascha E.J., Singh A., Sessler D.I., Kurz A. (2017). Relationship between Intraoperative Hypotension, Defined by Either Reduction from Baseline or Absolute Thresholds, and Acute Kidney and Myocardial Injury after Noncardiac Surgery: A Retrospective Cohort Analysis. Anesthesiology.

[18] Devereaux P.J., Beattie W.S., Choi P.T., Badner N.H., Guyatt G.H., Villar J.C., Cinà C.S., Leslie K., Jacka M.J., Montori V.M. (2005). How strong is the evidence for the use of perioperative beta blockers in non-cardiac surgery? Systematic review and meta-analysis of randomised controlled trials. BMJ.

[19] Barends C.R.M., Driesens M.K., Struys M., Visser A., Absalom A.R. (2020). Intranasal dexmedetomidine in elderly subjects with or without beta blockade: A randomised double-blind single-ascending-dose cohort study. Br. J. Anaesth..

[20] Yang S.S., Gelinas C., Yim E., Li M.M.J., Kardash K., Zhang M., Lipes J. (2022). Association of intraoperative dexmedetomidine use with postoperative hypotension in unilateral hip and knee arthroplasties: A historical cohort study. Can. J. Anaesth..

[21] Reddy V.S., Shaik N.A., Donthu B., Reddy Sannala V.K., Jangam V. (2013). Intravenous dexmedetomidine versus clonidine for prolongation of bupivacaine spinal anesthesia and analgesia: A randomized double-blind study. J. Anaesthesiol. Clin. Pharmacol..

[22] Shin H.J., Do S.H., Lee J.S., Kim T.K., Na H.S. (2019). Comparison of Intraoperative Sedation With Dexmedetomidine Versus Propofol on Acute Postoperative Pain in Total Knee Arthroplasty Under Spinal Anesthesia: A Randomized Trial. Anesth. Analg..

[23] Kim D., Jeong J.S., Park H., Sung K.S., Choi S.J., Gwak M.S., Kim G.S., Hahm T.S., Ko J.S. (2019). Postoperative pain control after the use of dexmedetomidine and propofol to sedate patients undergoing ankle surgery under spinal anesthesia: A randomized controlled trial. J. Pain Res..

[24] Hong B., Oh C., Jo Y., Chung W., Park E., Park H., Yoon S. (2021). The Effect of Intravenous Dexamethasone and Dexmedetomidine on Analgesia Duration of Supraclavicular Brachial Plexus Block: A Randomized, Four-Arm, Triple-Blinded, Placebo-Controlled Trial. J. Pers. Med..

[25] Beloeil H., Garot M., Lebuffe G., Gerbaud A., Bila J., Cuvillon P., Dubout E., Oger S., Nadaud J., Becret A. (2021). Balanced Opioid-free Anesthesia with Dexmedetomidine versus Balanced Anesthesia with Remifentanil for Major or Intermediate Noncardiac Surgery. Anesthesiology.

[26] Ma H., Wachtendorf L.J., Santer P., Schaefer M.S., Friedrich S., Nabel S., Ramachandran S.K., Shen C., Sundar E., Eikermann M. (2021). The effect of intraoperative dexmedetomidine administration on length of stay in the post-anesthesia care unit in ambulatory surgery: A hospital registry study. J. Clin. Anesth..

[27] Sin J.C.K., Tabah A., Campher M.J.J., Laupland K.B., Eley V.A. (2022). The Effect of Dexmedetomidine on Postanesthesia Care Unit Discharge and Recovery: A Systematic Review and Meta-Analysis. Anesth. Analg..

